# Impairment of Glucose Uptake Induced by Elevated Intracellular Ca^2+^ in Hippocampal Neurons of Malignant Hyperthermia-Susceptible Mice

**DOI:** 10.3390/cells13221888

**Published:** 2024-11-15

**Authors:** Arkady Uryash, Alfredo Mijares, Jose A. Adams, Jose R. Lopez

**Affiliations:** 1 Division of Neonatology, Mount Sinai Medical Center, Miami, FL 33140, USA; auryash@msmc.com (A.U.); jose.adams@msmc.com (J.A.A.); 2 Centro de Biofísica y Bioquímica, Instituto Venezolano de Investigaciones Científicas, Caracas 1020-A, Venezuela; mijaresa@gmail.com; 3 Department of Research, Mount Sinai Medical Center, Miami, FL 33140, USA

**Keywords:** intracellular Ca^2+^, glucose, hippocampal neurons, insulin resistance, dantrolene, SAR7334, BAPTA, PI3K/Akt, GLUT4

## Abstract

Malignant hyperthermia (MH) is a genetic disorder triggered by depolarizing muscle relaxants or halogenated inhalational anesthetics in genetically predisposed individuals who have a chronic elevated intracellular Ca^2+^ concentration ([Ca^2+^]_i_) in their muscle cells. We have reported that the muscle dysregulation of [Ca^2+^]_i_ impairs glucose uptake, leading to the development of insulin resistance in two rodent experimental models. In this study, we simultaneously measured the [Ca^2+^]_i_ and glucose uptake in single enzymatically isolated hippocampal pyramidal neurons from wild-type (WT) and MH-R163C mice. The [Ca^2+^]_i_ was recorded using a Ca^2+^-selective microelectrode, and the glucose uptake was assessed utilizing the fluorescent glucose analog 2-NBDG. The MH-R163C hippocampal neurons exhibited elevated [Ca^2+^]_i_ and impaired insulin-dependent glucose uptake compared with the WT neurons. Additionally, exposure to isoflurane exacerbated these deficiencies in the MH-R163C neurons, while the WT neurons remained unaffected. Lowering [Ca^2+^]_i_ using a Ca^2+^-free solution, SAR7334, or dantrolene increased the glucose uptake in the MH-R163C neurons without significantly affecting the WT neurons. However, further reduction of the [Ca^2+^]_i_ below the physiological level using BAPTA decreased the insulin-dependent glucose uptake in both genotypes. Furthermore, the homogenates of the MH-R163C hippocampal neurons showed an altered protein expression of the PI3K/Akt signaling pathway and GLUT4 compared with the WT mice. Our study demonstrated that the chronic elevation of [Ca^2+^]_i_ was sufficient to compromise the insulin-dependent glucose uptake in the MH-R163C hippocampal neurons. Moreover, reducing the [Ca^2+^]_i_ within a specific range (100–130 nM) could reverse insulin resistance, a hallmark of type 2 diabetes mellitus (T2D).

## 1. Introduction

Malignant hyperthermia (MH) is a potentially fatal pharmacogenetic disorder triggered in genetically susceptible individuals when exposed to depolarizing muscle relaxants or halogenated inhalational anesthetics [1]. MH is characterized by abnormal basal intracellular calcium concentrations [Ca^2+^]_i_ and excessive calcium release in response to specific triggers, primarily affecting skeletal muscle [2,3,4]. The underlying genetic factors associated with MH involve gene mutations, such as ryanodine receptor isoform 1 (RyR1), calcium voltage-gated channel subunit alpha1 S (CACNA1S), and STAC3 [1,5].

While the role of skeletal muscle in MH is well-established [1,2,3,4,6], little is known about how these mutations impact neuronal function. Recent findings from our laboratory revealed significantly elevated basal [Ca^2+^]_i_ levels in the cortical neurons of MH-R163C mice, which were further exacerbated by isoflurane exposure [7]. This suggests that MH mutations may also disrupt neuronal calcium homeostasis and potentially affect critical cellular processes.

Despite representing only 2% of the body weight, the brain consumes approximately 20% of the body’s total glucose [8]. Neurons rely heavily on glucose for energy, and its uptake is regulated by glucose transporters (GLUTs), which ensure a consistent supply of glucose to meet the brain’s high metabolic demands [9].

The interaction between calcium signaling and glucose metabolism is complex, and previous studies in muscle cells showed that the sustained elevation of [Ca^2+^]_i_ can impair PI3K/Akt/SA160 signaling and modify GLUT4 expression and subcellular distribution [6,10]. Similarly, calcium has been implicated in regulating the insulin-mediated glucose uptake in adipocytes [11,12]. However, whether dysregulated calcium levels in neurons can impair glucose uptake remains unclear.

We hypothesize that the chronic elevation of [Ca^2+^]_i_ in MH-R163C neurons disrupts insulin-dependent glucose uptake, impairing neuronal glucose metabolism. The objectives of this study were threefold. First, we aimed to investigate whether hippocampal neurons carrying the MH-R163C mutation exhibit altered insulin-dependent glucose uptake compared with WT neurons. Second, we sought to evaluate the effects of isoflurane and calcium-lowering agents on the glucose uptake in MH-R163C neurons to understand how these interventions modulate impaired glucose metabolism. Finally, we aimed to assess the expression levels of critical proteins involved in glucose metabolism, including PI3K/Akt/SA160 and GLUT4, in MH-R163C neurons to elucidate the molecular mechanisms underlying the observed glucose dysregulation.

Our study demonstrated that [Ca^2+^]_i_ dysregulation is a critical factor that affected the neuronal insulin-dependent glucose uptake, which provided insights into potential therapeutic strategies for addressing glucose dysmetabolism in MH.

## 2. Materials and Methods

### 2.1. Experimental Model

The experiments were conducted in male and female wild-type C57BL/6J (WT) mice and heterozygous knock-in mice of C57BL/6 for the RyR1 variant, which led to the amino acid change p.R163C in the RyR1 protein (MH-R163C) at 3 to 6 months of age. All animals were housed in ventilated cages with equal mice (*n* = 6), which ensured standard pathogen-free conditions at 23 °C, with a regular 12:1 h light–dark cycle. The mice received standard mouse food and water ad libitum and were kept in the Mount Sinai Medical Center vivarium.

### 2.2. Hippocampal Pyramidal Neuron Cultures

It is widely recognized that the dysregulation of neuronal intracellular calcium homeostasis can contribute to the development of neurodegenerative disorders, such as Alzheimer’s disease, and cognitive impairments [13,14]. Given the critical role of the hippocampus in cognitive functions, we extended our previous work in MH-R163C cortical neurons [7] to determine how the dysregulation of intracellular Ca^2+^ might affect this brain region, where all three RyR isoforms—RyR1, RyR2, and RyR3—are known to be expressed, particularly in the cerebellum, hippocampus, and cerebral cortex [15,16,17]. As previously reported, MH-R163C and WT pyramidal hippocampal neurons were obtained from female and male mice using a modified method [18,19]. The hippocampus was carefully isolated and placed in ice-cold Hibernate A medium (BrainBits, Springfield, IL, USA) supplemented with B27 and glutamine (Thermo Fisher, Waltham, MA, USA). Subsequently, the hippocampal tissue was finely diced into small fragments, and the enzymatic dissociation of the cells was carried out in a Hibernate A/B27 solution that contained 2 mg/mL Papain (Worthington, Columbia, NJ, USA). The digested tissue was transferred to a papain-free medium and triturated using a fire-polished Pasteur pipette. Following trituration, the tissue was gently layered on the surface of an OptiPrep 1.32 gradient (Sigma-Aldrich, St. Lois, MO, USA) and subjected to centrifugation at 800× *g* for 15 min. Subsequent centrifugation at 200× *g* for 3 min resulted in the formation of a pellet, which was gently resuspended in Neurobasal A medium supplemented with 0.5 mM glutamine, 10 ng/mL fibroblast growth factor-2, 10 ng/mL brain-derived neurotrophic factor, 1% penicillin, and 1% streptomycin. The enzyme-isolated neurons were then seeded in 6- or 96-well plates coated with poly-D-lysine and laminin (Corning Incorporated, Tewksbury, MA, USA) and allowed to incubate for 2 h at 37 °C under conditions of 5% CO_2_ and 10% O_2_. After this incubation period, the unattached cells were removed, and the culture medium was replaced with Neurobasal A supplemented with 1% penicillin, 1% streptomycin, 1% glutamine, 5 ng/mL basic fibroblast growth factor, and 3 mM/L L-carnitine. The experiments were carried out on cultured cells incubated for 6 days at 37 °C in an environment of 5% CO_2_ and 95% air.

### 2.3. Glucose Determinations

Blood samples (5 μL) were taken from the tail veins of the WT and MH-R163C mice for glucose analysis using direct flow or gentle massaging techniques [10]. Glucose measurements were performed using a handheld glucometer (AlphaTRAK^®^ glucose meter, Abbott Animal Health, Abbott Park, IL, USA) [10]. It is worth noting that all MH-R163C mice included in this study consistently exhibited blood glucose levels greater than 250 mg/dL over three consecutive tests.

### 2.4. Ca^2+^ Selective Microelectrodes

Double-barreled, Ca^2+^-selective microelectrodes were prepared as previously described [2]. They were prepared from thin-walled borosilicate glass capillaries with outside diameters (ODs) of 1.2 mm and 1.5 mm (PB150F-4, World Precision Instruments, Sarasota, FL, USA). The tip of a 1.5 mm barrel OD was silanized by exposure to vaporized dimethyldichlorosilane (Sigma-Aldrich, St. Lois, MO, USA) and filled with a liquid neutral ion carrier based on ETH 129 for Ca^2+^ (Sigma-Aldrich, St. Lois, MO, USA) and the remaining portion of the barrel was backfilled with pCa7. Subsequently, the second barrel (1.2 mm OD) was filled with 3 M KCl a few minutes before recording. Specific potentials were recorded at a frequency of 1000 Hz using AxoGraph software (version 4.6; Molecular Devices, San Jose, CA, USA) and stored on a computer for subsequent analysis. To ensure precision in reading, each Ca^2+^-selective microelectrode was individually calibrated before and after measurements, following the published protocols [2,20]. If the calibration curves of both calibrations differed by more than 3 mV, the data obtained from that specific microelectrode were discarded [2,19,20].

### 2.5. Exposure to Isoflurane In Vitro

Cultured neurons were exposed to 1.5% isoflurane administered in a mixture of 95% air and 5% CO_2_ using a precisely calibrated anesthesia vaporizer (Kent Scientific, Torrington, CT, USA). This vaporizer was connected to a jacketed reservoir containing a Ringer-Locke solution maintained at 37 °C. The solutions were delivered to the experimental chamber using a speed-variable peristaltic pump (Harvard Apparatus, Holliston, MA, USA) through an unsharpened pipette placed over the recorded neuron. Perfusate samples were analyzed using an HPLC method [17] to confirm the final concentration of the isoflurane.

### 2.6. Single-Neuron Fluorescent 2-NBDG Uptake

To evaluate the glucose uptake, pyramidal hippocampal neurons isolated from the MH-R163C and WT mice were incubated in a glucose-free Ringer-Locke solution containing 300 µM of the fluorescent glucose analog 2-(N-(7-Nitrobenz-2-oxa-1,3-diazol-4-yl)amino)-2-deoxyglucose (2-NBDG) (Thermo Fisher Scientific, Carlsbad, CA, USA) for 45 min [10]. After the incubation, the neurons were thoroughly washed with a glucose-free Ringer-Locke solution to remove excess dye and then mounted on an inverted microscope stage (Zeiss, White Plains, NY, USA) equipped with a 40× water immersion objective (FWD = 2.5 mm) for fluorescence recording. The neurons were incubated with 100 µM insulin for 45 min, and fluorescence recording was initiated after reintroducing normal Ringer-Locke solution (containing glucose) into the bath. We used insulin containing meta-cresol as an excipient (Sanofi Pharmaceuticals, Township, NY, USA) instead of 4-chloro-m-cresol to avoid potential experimental artifacts, given the increased sensitivity of MH-susceptible hippocampal neurons to 4-chloro-m-cresol [21,22].

Fluorescence measurements were performed using an excitation wavelength of 480 nm and an emission wavelength of 550 nm. To minimize potential artifacts associated with uneven dye loading, intracellular compartmentalization, or dye leakage over time, glucose uptake was evaluated by continuously measuring the rate of 2-NBDG uptake (fluorescence intensity as a function of time) in a single hippocampal neuron rather than relying on end-point measurements or fluorescence signals from a group of cells. The uptake rate was determined by analyzing the slope of the 2-NBDG fluorescence intensity signal during the observation period using GraphPad Prism 9.0 software (GraphPad Software, Boston, MA, USA), and the results were normalized to WT control values. Representative recordings of these measurements are found in the Supplementary Materials as Supplemental Figure S1.

### 2.7. Measurements of Neuronal [Ca^2+^]_i_ and Glucose Uptake in a Single Neuron

Neuronal [Ca^2+^]_i_ and insulin-dependent glucose uptake were simultaneously measured in single MH-R163C and WT hippocampal neurons. A single dye-loaded neuron (identified by its fluorescence) was subsequently impaled with a Ca^2−^ selective microelectrode under microscope observation, and measurements were recorded after the treatment with insulin (see Section 2.6). Signals were stored on a computer for future analysis, and the criteria for successful neuronal measurements of [Ca^2+^]_i_ and glucose uptake were previously described [7,23]. Measurements were performed before and after incubation in (i) a Ca^2+^-free solution, (ii) 10 µM BAPTA, (iii) 1 µM SAR7334, and (iv) 20 µM dantrolene, as well as (v) exposure to 1.5% isoflurane. The experiments were conducted at 37 °C.

### 2.8. Protein Expressions

The WT and MH-R163C mice were anesthetized (100 mg/kg of ketamine and 5 mg/kg of xylazine), euthanized with CO_2,_ and then their hippocampuses were harvested. Total protein extraction was performed using Millipore enzyme buffer with 0.5% Triton-X-100 (Sigma-Aldrich, St. Lois, MO, USA). The total protein concentration was determined, and then the protein samples were processed with gel electrophoresis and transferred to a cellulose membrane as previously described [10]. Individual membrane strips were prepared according to the molecular weights of the proteins of interest and then incubated with primary and secondary antibodies. The following antibodies were used: PI3K, pPI3K, Akt, pAkt, AS160, pA160, and GLUT4 (Abcam, Cambridge, MA, USA). Actin was used as a protein loading control (Abcam, Cambridge, MA, USA). The corresponding protein size was determined based on the Bio-Rad protein standard via fluorescent scanning. Target protein values (s) were normalized to the loading control using the housekeeping protein β-actin [10].

### 2.9. Solutions

The Ringer-Locke solution contained (in mM) 135 NaCl, 5 KCl, 2 CaCl_2_, 1 MgCl_2_, 5 glucose, 3.6 NaHCO_3_ (pH 7.4). The Ca^2+^-free solution had the same composition as the Ringer-Locke solution, but Ca^2+^ was omitted, and 1 mM EGTA and 2 mM MgCl_2_ were added. The glucose-free Ringer-Locke’s solution was prepared by omitting glucose. Dantrolene (20 µM) (Sigma-Aldrich, St. Lois, MO, USA), 5,5-dimethyl bis (o-aminophenoxy) ethane-N,N,N,N tetraaccetic acetoxymethyl ester (BAPTA) (10 µM) (Abcam, Waltham, MA, USA), and SAR7334 (1 µM) (Biotechne Tocris, Minneapolis, MN, USA) were prepared by adding concentrated stocks in dimethylsulfoxide to the Ringer-Locke solution. To prevent spontaneous neuronal firing that could interfere with the recording of the [Ca^2+^]_i_, the Ringer-Locke solution was supplemented with 1.5 µM tetrodotoxin (Sigma-Aldrich, St. Lois, MO, USA) [23]. The Ringer-Locke solution was aerated with a mixture of 95% O_2_ and 5% CO_2_ for all experiments (37 °C).

### 2.10. Statistical Analysis

Data are presented as the mean ± standard deviation. We excluded all data from neurons that showed a resting membrane potential of less than −65 mV. We used the D’Agostino and Pearson test to determine whether the samples were normally distributed. We compared the experimental values using a one-way and two-way analysis of variance (ANOVA) and Tukey’s post hoc test (GraphPad Software Prism 9.0, Boston, MA, USA). In addition, the Mann–Whitney test was used for samples that did not follow a normal distribution (Python Software Foundation v3.11.4, Wilmington, DE, USA). A *p*-value < 0.05 was considered significant. *n*: equal to the number of successful measurements, and *n_mice_*: equal to the number of mice used experimentally.

## 3. Results

### 3.1. Elevated [Ca^2+^]_i_ and Diminished Insulin Glucose Uptake

**Figure 1:**
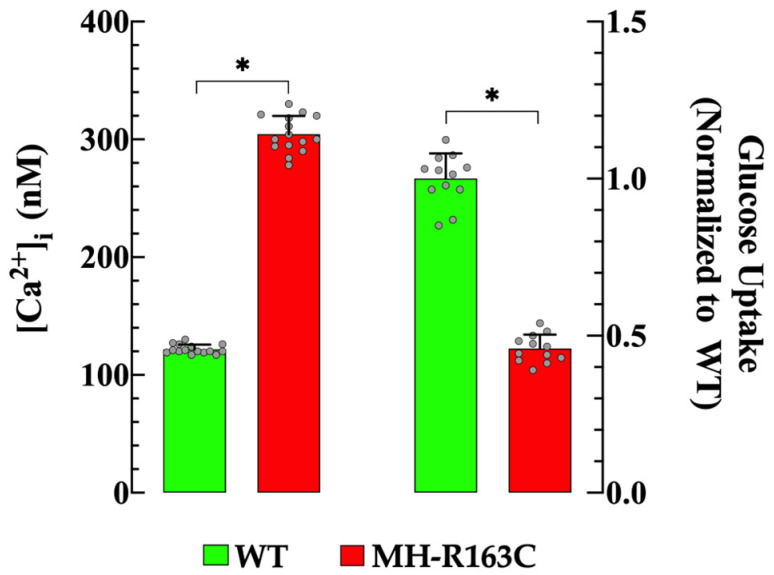
Resting [Ca^2+^]_i_ and insulin-dependent glucose uptake in the WT and MH-163C neurons. Values are expressed as the mean ± SD. The grey circles represent individual experimental values recorded under each condition. *n =* 12–15, *n_mice_* = 8. * denotes *p* < 0.05.

We have recently provided evidence that chronically elevated levels of [Ca^2+^]_i_ decreased glucose uptake in isolated skeletal muscle [6,10]. We further explored this relationship by simultaneous measurements of the [Ca^2+^]_i_ and insulin-dependent glucose uptake in MH-R163C hippocampal neurons. We confirmed a significant elevation in [Ca^2+^] I in the MH-R163C neurons compared with the WT neurons [7] (Figure 1, left panel and Supplemental Figure S1). Furthermore, we revealed, for the first time, a significant decrease in insulin-mediated glucose uptake compared with the WT neurons (Figure 1, right panel, and Supplemental Figure S1). In the WT neurons, the hippocampal neuron [Ca^2+^]_i_ was 122 ± 4 nM (*n* = 15), while in the MH-R163C neurons, it was 304 ± 15 nM (*n* = 15, *p* < 0.05 compared with the WT neurons). Furthermore, our investigation revealed a 2.6-fold decrease in insulin-stimulated glucose in the MH-R163C neurons compared with the WT neurons (*p* < 0.05 compared with the WT neurons) (Figure 1, right panel and Supplemental Figure S1).

### 3.2. Effects of Extracellular Ca^2+^ Removal on [Ca^2+^]_i_ and Glucose Uptake

**Figure 2:**
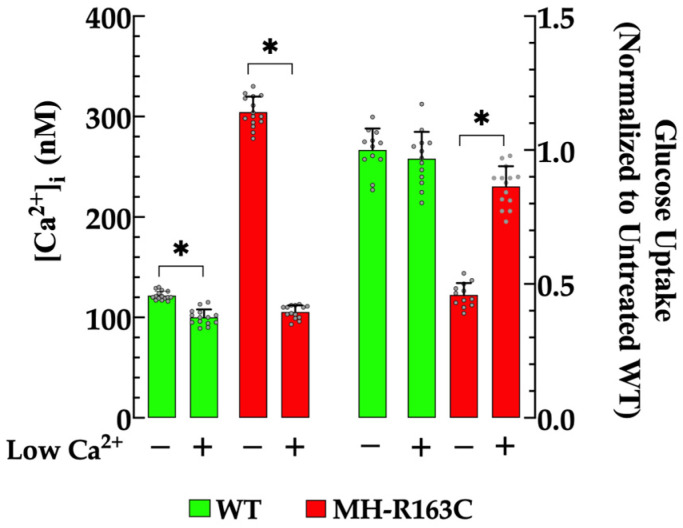
Effects of the [Ca^2+^]_e_-free solution on the resting [Ca^2+^]_I_ and insulin-dependent glucose uptake. Values are expressed as the mean ± SD. The grey circles represent individual experimental values recorded under each condition. *n =* 12–15, *n_mice_* = 9. * denotes *p* < 0.05.

In separate experimental setups, we examined the effect of Ca^2+^-free solution on the [Ca^2+^]_i_ and insulin-dependent glucose uptake in the WT and MH-R163C hippocampal neurons. The omission of Ca^2+^ in the Ringer-Locke solution (as described in the Section 2) significantly reduced the [Ca^2+^]_i_ in both genotypes and increased the insulin-stimulated glucose uptake only in the MH-R163C neurons. The exposure of the MH-R163C neurons to the Ca^2+^-free solution reduced the [Ca^2+^]_i_ to 105 ± 6 nM (*n* = 14, *p* < 0.05 compared with the untreated MH-R163C neurons) (Figure 2, left panel), and caused a significant increase (1.9-fold) in the insulin-dependent glucose uptake (*p* < 0.05 compared with the untreated MH-R163C neurons) (Figure 2, right panel). On the other hand, the incubation of the WT neurons with a Ca^2+^-free solution also decreased the [Ca^2+^]_i_ to 100 ± 8 nM (*n* = 14, *p* < 0.05 compared with the untreated WT neurons) (Figure 2 left panel), but it did not induce a significant alteration in the neuronal glucose uptake (*p* = 0.73) (Figure 2 right panel).

### 3.3. Effects of BAPTA on [Ca^2+^]_i_ and Glucose Uptake

**Figure 3:**
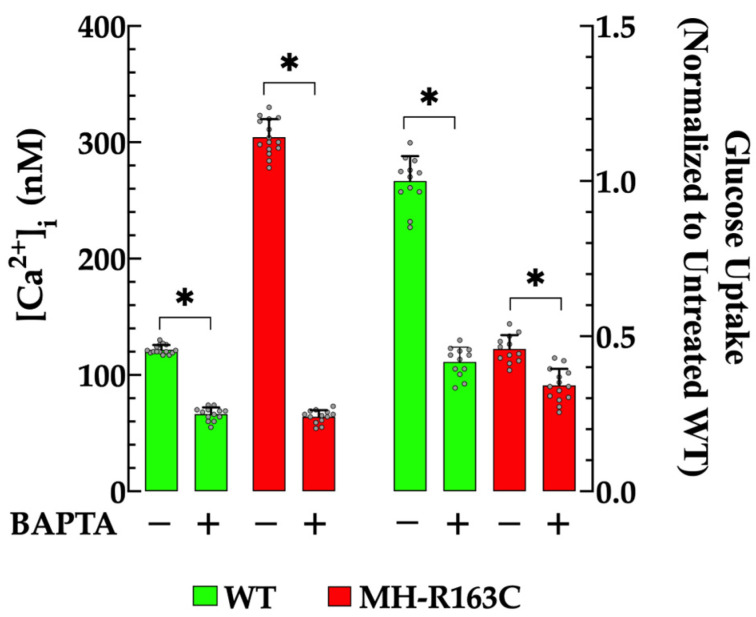
Effects of BAPTA on the resting [Ca^2+^]_I_ and insulin-dependent glucose uptake. Values are expressed as the mean ± SD. The grey circles represent individual experimental values recorded under each condition. *n =* 12–15, *n_mice_* = 5. * denotes *p* < 0.05.

Subsequently, we investigated the effect of increasing the intracellular Ca^2+^ buffering capacity using the Ca^2+^ chelator BAPTA-AM [22] on the [Ca^2+^]_i_ and glucose uptake in the WT and MH-R163C hippocampal neurons. The treatment of the WT neurons with 10 µM BAPTA for 30 min resulted in a reduction in [Ca^2+^]_i_ to 66 ± 4 nM (*n* = 13, *p* < 0.05 compared with the untreated WT neurons), while in the MH-R163C neurons, the [Ca^2+^]_i_ decreased to 64 ± 6 nM (*n* = 15, *p* < 0.05 compared with the untreated MH-R163C neurons) (Figure 3, left panel). Notably, the glucose uptake was reduced 2.4-fold in the WT neurons (*p* < 0.05 compared with the untreated WT neurons) and 1.4-fold in the MH-R163C neurons (*p* < 0.05 compared with the untreated MH-R163C neurons) (Figure 3, right panel).

### 3.4. Blocking TRPC Channels Affected [Ca^2+^]_i_ and Glucose Uptake

**Figure 4:**
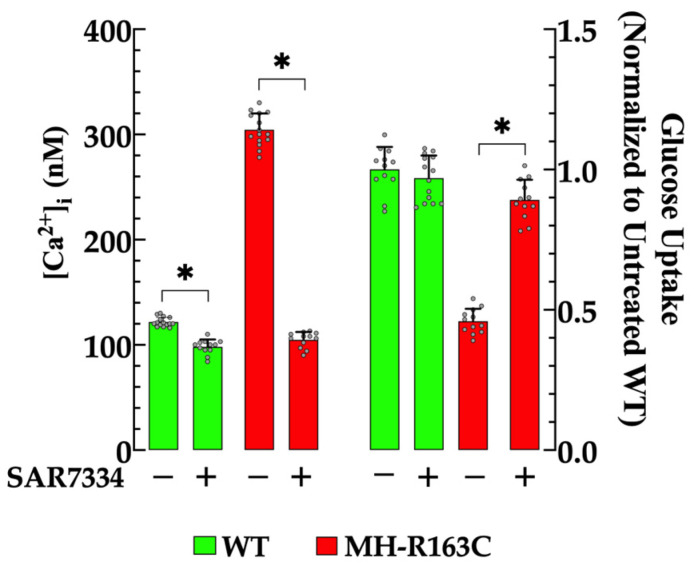
Regulation of the [Ca^2+^]_i_ and insulin-dependent glucose transport by SAR7334. Values are expressed as the mean ± SD. The grey circles represent individual experimental values recorded under each condition. *n =* 12–15, *n_mice_* = 8. * denotes *p* < 0.05.

To further examine the relationship between the [Ca^2+^]_i_ and insulin-stimulated glucose uptake, the WT and MH-R163C hippocampal neurons were treated with SAR7334, a specific blocker of TRPC3 and TRPC6 channels [24]. We have previously demonstrated the contributions of TRPC3 and 6 to elevated intracellular [Ca^2+^] in MH muscle at rest and during an episode of MH [2,25]. Incubation with 1 μM SAR7334 resulted in a significant reduction in the [Ca^2+^]_i_ in both genotypes. Specifically, in the WT neurons, [Ca^2+^]_i_ decreased to 99 ± 7 nM (*n* = 12, *p* < 0.05 compared with the untreated WT neurons), while in the MH-R163C neurons, it decreased to 105 ± 7 nM (*n* = 12, *p* < 0.05 compared with the untreated MH-R163C neurons) (Figure 4, left panel). Furthermore, the SAR7334 treatment led to a 1.9-fold increase in the glucose uptake in the MH-R163C neurons (*p* < 0.05 compared with the untreated MH-R163C neurons), while it did not significantly affect the glucose uptake in the WT neurons (*p* = 0.72) (Figure 4, right panel).

### 3.5. Effects of Dantrolene on [Ca^2+^]_i_ and Glucose Uptake

**Figure 5:**
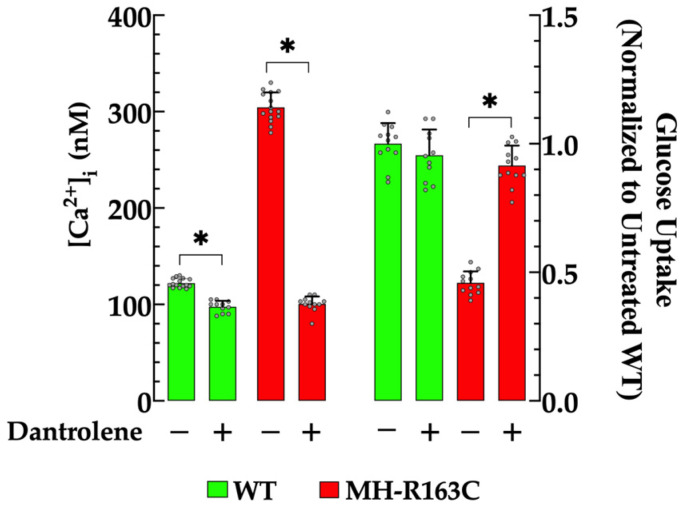
Modulation of the [Ca^2+^]_i_ and insulin-dependent glucose uptake by dantrolene. Values are expressed as the mean ± SD. The grey circles represent individual experimental values recorded under each condition. *n =* 11–15, *n_mice_* = 7. * denotes *p* < 0.05.

Dantrolene is a well-known agent used clinically to reduce spasticity or treat malignant hyperthermia in susceptible patients and experimental models [26,27,28]. At the cellular level, it blocks Ca^2+^ leakage from the ryanodine receptor and mitigates the Ca^2+^ influx into excitable cells [29,30]. The incubation with 20 µM dantrolene resulted in a significant reduction in the neuronal [Ca^2+^]_i_ in both genotypes and an increase in the glucose uptake in the MH-R163C neurons. Specifically, in the WT neurons, dantrolene decreased the [Ca^2+^]_i_ to 98 ± 6 nM (*n* = 11, *p* < 0.05 compared with untreated WT neurons), while in the MH-R163C neurons, it decreased to 101 ± 8 nM (*n* = 12, *p* < 0.05 compared with the untreated MH-R163C neurons) (Figure 5, left panel). Furthermore, the dantrolene treatment significantly increased the glucose uptake 2-fold in MH-R163C neurons (*p* < 0.05 compared with untreated MH-R163C neurons), while it did not significantly affect the glucose uptake in the WT neurons (*p* = 0.55) (Figure 5, right panel). A higher concentration of dantrolene > 30 µM further reduced the [Ca^2+^]_i_ and inhibited the insulin-dependent glucose uptake in both genotypes.

### 3.6. Effects of Isoflurane on [Ca^2+^]_i_ and Glucose Uptake

**Figure 6:**
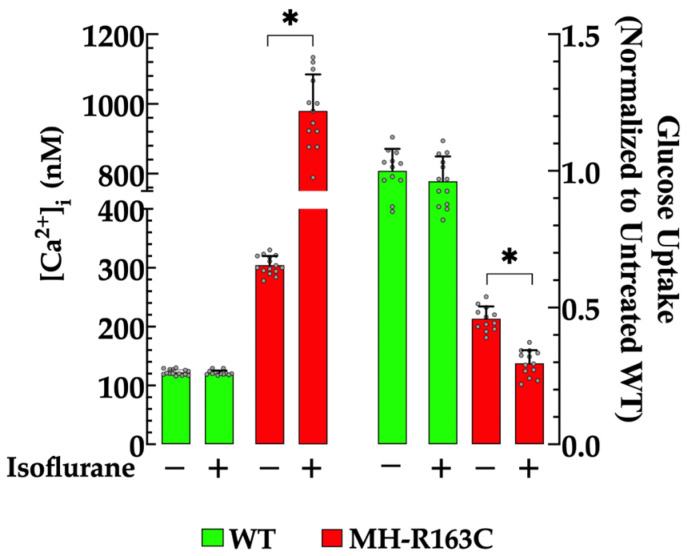
Isoflurane-induced changes in the [Ca^2+^]_i_ and insulin-mediated glucose uptake in the MH-R163 neurons. Values are expressed as the mean ± SD. The grey circles represent individual experimental values recorded under each condition. *n =* 12–15, *n_mice_* = 8. * denotes *p* < 0.05.

Our previous report demonstrated that isoflurane, a well-known trigger anesthetic for MH [1], increased [Ca^2+^]_i_ in cortical neurons in mice susceptible to MH [7]. We corroborated that isoflurane 1.5% further escalated the [Ca^2+^]_i_ and found that it significantly exacerbated already impaired insulin-dependent glucose uptake in the MH-R163C neurons. Specifically, the [Ca^2+^]_i_ was elevated to 980 ± 104 nM (*n* = 13, *p* < 0.05 compared with the untreated MH-R163C neurons) (Figure 6 left panel), and the glucose uptake was significantly reduced 1.5-fold (*n =* 13, *p* < 0.05 compared with the untreated MH-R163C neurons) (Figure 6 right panel). In contrast, the WT neurons exhibited no significant changes in either [Ca^2+^]_i_ (122 ± 3 nM, *n* = 15, *p* = 0.99) or glucose uptake after exposure to isoflurane (*n* = 13, *p* = 0.58) (Figure 6 left and right panels, respectively).

### 3.7. Effect of Dantrolene on Blood Glucose Levels

**Figure 7:**
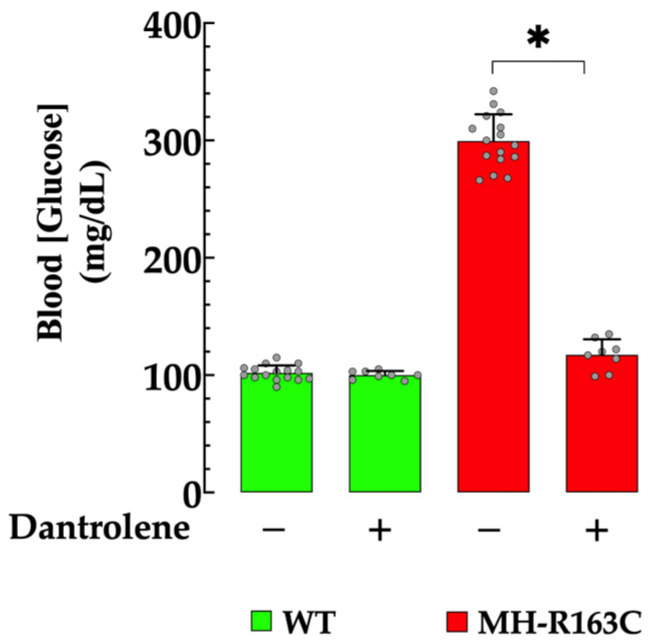
Dantrolene reduced the blood glucose levels in the MH-R163C mice. Values are expressed as the mean ± SD. The grey circles represent individual experimental values recorded under each condition. *n =* 8–16, *n_mice_* = 8. * denotes *p* < 0.05.

**Figure 8:**
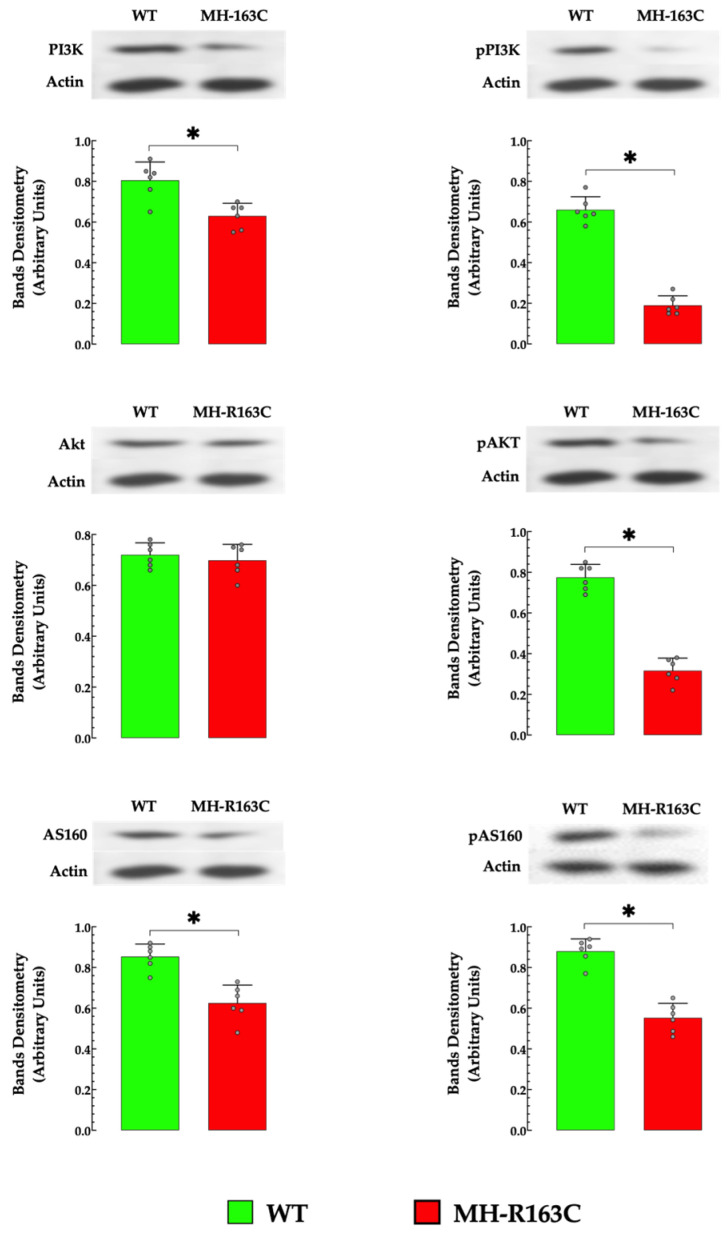
Abnormal expressions of proteins of the insulin signaling pathway in the hippocampal neurons. Representative Western blot and densitometric analysis of PI3K, Akt, and AS160 protein expression in hippocampal homogenates. The grey circles represent individual experimental values recorded under each condition. The data were normalized to actin and expressed as the mean ± S.D. *n* = 4–6 per condition and *n_mice_* = 5. * denotes *p* < 0.05.

We have reported altered glucose homeostasis in patients and rodents with chronic elevation of [Ca^2+^]_i_ in skeletal muscle [6,10]. The mean basal blood glucose value in the fasting WT mice was 102 ± 6 mg/dL (*n* = 16), while in the MH-R163C mice, it was 299 ± 23 mg/dL (*n* = 16) (*p* < 0.05 compared with the WT mice) (Figure 7). The WT and MH-R163C mice received dantrolene (1 mg/kg, IP day) for 7 consecutive days [19], and then the fasting blood glucose was determined again. Interestingly, in the *MH-R163C* mice, the dantrolene treatment led to a significant reduction in the blood glucose values to 117 ± 13 mg/dL (*n* = 8) (*p* < 0.001 compared with the untreated *MH-R163C* mice) (Figure 8). No effect on the blood glucose values was observed in the WT mice (100± 4 mg/dL, *n*= 8, *p* = 0.99).

### 3.8. Hippocampal Protein Expression

**Figure 9:**
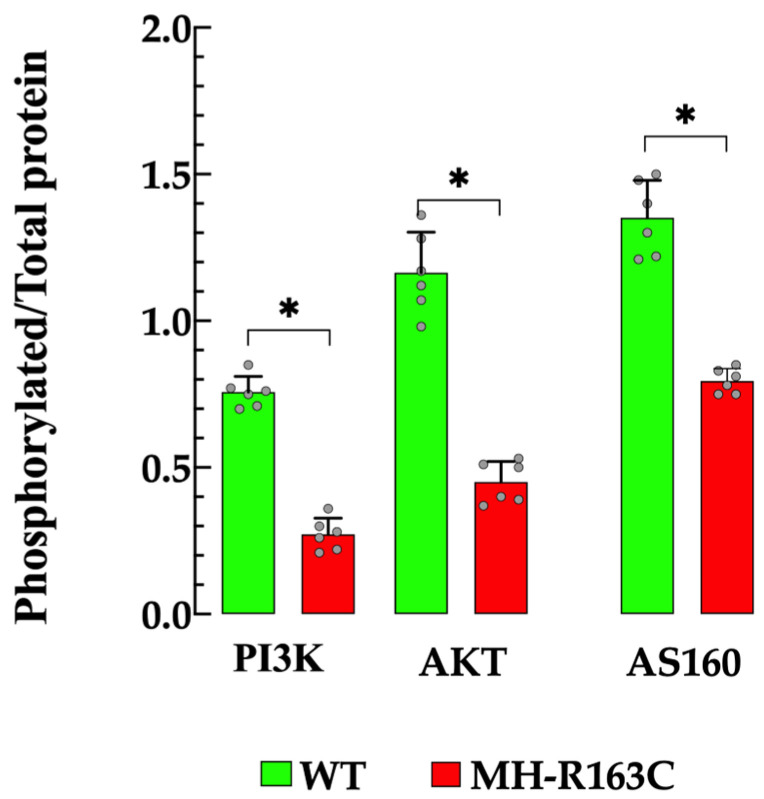
Phosphorylated/total protein ratio. The grey circles represent the individual experimental values recorded under each condition. The data were normalized to actin and expressed as the mean ± S.D. *n* = 4–6 per condition and *n_mice_* = 5. * denotes *p* < 0.05.

**Figure 10:**
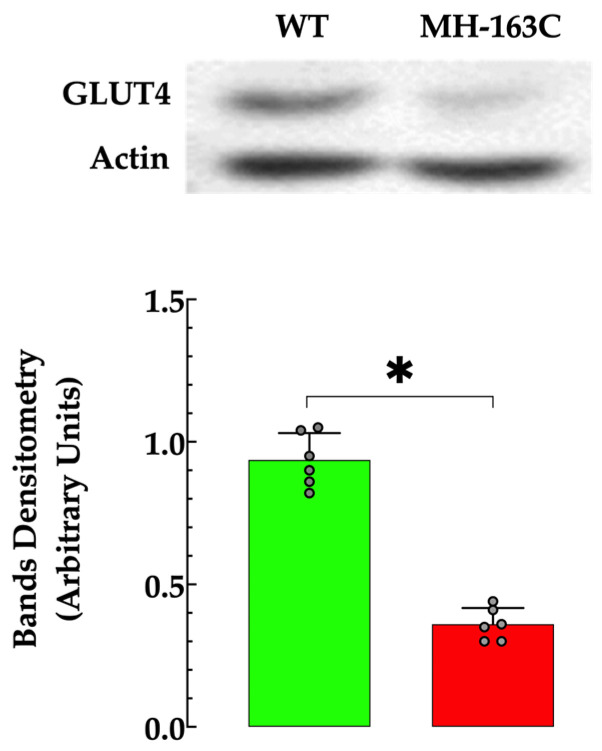
Decreased expression of GLUT4 in the hippocampus MH-R163C neurons. The data were normalized to actin, and the values are expressed as the mean ± SD. The grey circles represent individual experimental values recorded under each condition. *n =* 6, *n_mice_* = 5. * denotes *p* < 0.05.

Hippocampal homogenates from MH-R163C mice showed significantly altered insulin signal transduction processes downstream of PI3K, pPI3K, pAKT, AS160.pAS160, and GLUT4 protein expression compared with the WT (Figure 8). This alteration was evident as a decrease in the PI3K protein expression by 1.3-fold, pPI3K by 3.9-fold, pAkt by 2.4-fold, AS160 by 1.3-fold, and pAS160 by 2.3-fold in the MH-R163C compared with the WT (Figure 8). However, no significant changes were observed in the expression levels of Akt. Furthermore, decreases in the ratio of the phosphorylated/total protein for PI3K (64%), Akt (61%), and AS160 (41%) was observed in the MH-R163C neurons compared with the WT (Figure 9). Additionally, the MH-R163C mice exhibited significantly lower expression of the glucose transporter GLUT4 (2.3-fold) compared with the WT mice (Figure 10).

## 4. Discussion

The present study confirmed the abnormal [Ca^2+^]_i_ and provided the first evidence of aberrant insulin-mediated signaling in hippocampal neurons isolated from a rodent MH-R163C model.

The key findings include the following:

The MH-R163C hippocampal neurons revealed a significant increase in the [Ca^2+^]_i_ and a decrease in the insulin-mediated glucose uptake compared with the WT neurons.

The neurons exposed to the Ca^2+^-free solution significantly decreased the [Ca^2+^]_I_ in both genotypes, with an increase in insulin-mediated glucose uptake observed only in the MH-R163C neurons.

Chelating the [Ca^2+^]_i_ with BAPTA resulted in a reduction in the [Ca^2+^]_i_ and a glucose uptake in both genotypes.

SAR7334, a TRPC3 and TRPC6 channel blocker, significantly reduced the [Ca^2+^]_i_ in both genotypes. This was accompanied by an increased glucose uptake in the MH-R163C neurons without a detectable effect in the WT neurons.

Dantrolene, an inhibitor of intracellular Ca^2+^ release and influx, significantly reduced the neuronal [Ca^2+^]_i_ in both genotypes and increased the glucose uptake in the MH-R163C neurons. No noticeable effect was found in the WT mice.

Isoflurane, a trigger agent for the episode of MH, further elevated the [Ca^2+^]_i_ and significantly worsened the already impaired insulin-dependent glucose uptake in the MH-R163C neurons. No discernible effect was observed in the WT mice.

The treatment of mice with dantrolene significantly reduced the blood glucose values in the MH-R163C mice, while no effect was observed in the WT mice.

The hippocampal homogenates from the MH-R163C mice exhibited significantly altered protein expressions of PI3K, pPI3K, pAkt, AS160, pAS160, and GLUT4 compared with the WT mice.

### 4.1. Abnormal Neuronal Intracellular [Ca^2+^]

Intracellular Ca^2+^ represents a universal signaling molecule, orchestrating countless cellular processes, such as muscle contraction, neurotransmission, hormone secretion, metabolism, and cell growth. The intracellular Ca^2+^ concentration in excitable cells is tightly regulated around 100–120 nM [4,7,19,20,31], an equilibrium that is maintained through the spatio-temporal interplay of various mechanisms, such as the Ca^2+^ influx that occurs through various channels and transporters, including voltage-gated Ca^2+^ channels, ligand-gated channels, and receptor-operated channels, and Ca^2+^ uptake by the sarcoendoplasmic reticulum and mitochondria, as well as the plasma membrane calcium ATPase and the sodium/calcium (Na^+^/Ca^2+^) exchanger [32,33]. Disruptions in the delicate balance of intracellular Ca^2+^ dynamics have recently emerged as a critical factor contributing to the onset and progression of insulin resistance in T2D [6,10,11]. We found a sustained elevation of [Ca^2+^]_i_ in MH-163C hippocampal neurons (304 nM), which could be related to increased RyR leaks and increased calcium influx mediated by upregulated TRPC3 and 6 channels active at negative membrane potentials, as previously reported in MH muscle cells [2,25]. Interestingly, our study revealed a significantly diminished intracellular calcium content within the sarcoplasmic/endoplasmic reticulum (SER) of the MH-R163C hippocampal neurons compared with the WT neurons (Supplemental Figure S2). This observation supports the hypothesis that an increased resting ryanodine receptor Ca^2+^ leak occurs in MH-R163C hippocampal neurons, leading to reduced SER Ca^2+^ loading. As previously described, this reduction in SER calcium content can provoke the upregulation of voltage-independent TRPC3 and TRPC6 channels [7], resulting in an increased Ca^2+^ influx. This mechanism may contribute to the altered calcium homeostasis observed in MH-R163C neurons and can have broader implications for understanding the pathophysiology associated with this mutation. An increase in Ca^2+^ entry depending on the calcium content of the intracellular store has been described in intact adult and fetal mammalian skeletal muscle [34].

Studies demonstrated that elevated [Ca^2+^]_i_ can cause mitochondrial dysfunction, a critical factor in the development of insulin resistance, as observed in T2D [35,36]. Excess Ca^2+^ influx into mitochondria, resulting from elevated [Ca^2+^]_i_, can lead to mitochondrial Ca^2+^ overload, impaired ATP production [37,38], and increased production of reactive oxygen species [39]. This oxidative stress damages critical mitochondrial components, including proteins, lipids, and mitochondrial DNA, leading to further mitochondrial dysfunction, insulin resistance, and eventual cell death [40].

Isoflurane, a widely recognized trigger for MH [1,3,41], exacerbated the elevation of [Ca^2+^]_i_ in the MH-R163C neurons, mirroring previous findings in skeletal muscle [3]. These exciting results challenge the conventional understanding of the pathophysiology of MH, which historically centered on intracellular calcium dysregulation within skeletal muscle [4,21,42,43]. Our study revealed that the MH-R163C hippocampal neurons exhibited aberrant [Ca^2+^]_i_ that was consistent with those previously documented in cortical neurons [7], providing further support for the existence of extra-muscular alterations related to susceptibility to MH.

It should be noted that disruptions in neuronal [Ca^2+^]_i_ have been recognized as a significant factor in neurological conditions, such as Alzheimer’s and Parkinson’s disease, aging [13,14,44,45], and cognitive decline [14]. These findings suggest that individuals susceptible to malignant hyperthermia may be predisposed to conditions such as Alzheimer’s and Parkinson’s disease, as well as some degree of cognitive impairment. This clinical aspect has not been thoroughly explored and should be deeply investigated.

### 4.2. Calcium Signaling in Insulin-Dependent Glucose Uptake

Glucose constitutes the primary energy source for brain metabolism under normal physiological conditions. Neurons demand a continuous stream of glucose because they cannot store it [46]. The transmembrane transport of glucose is facilitated by specialized membrane transporters known as glucose transporter proteins (GLUTs) [46]. Ca^2+^ plays a crucial role in insulin-mediated cell signaling within target tissues [47]. Insulin triggers a localized and transient elevation [Ca^2+^]_i_ near the plasma membrane in skeletal muscle cells [48], cardiomyocytes [49], and cultured myotubes [50]. Transient elevation in [Ca^2+^]_i_ triggered by insulin lasts for a few minutes and depends mainly on the extracellular Ca^2+^ influx through L-type calcium channels [51] and the Na^+^/Ca^2+^ exchanger [52], facilitating Ca^2+^ influx into the cytosol. Subsequently, this influx activates the RyR and inositol triphosphate receptors, leading to Ca^2+^ release from intracellular stores [53].

Although transient increases in [Ca^2+^]_i_ induced by insulin can stimulate glucose uptake in target tissues [47,54], a chronic and aberrant elevation of [Ca^2+^]_i_ can have a significant impairment for insulin-dependent glucose uptake and the context of insulin resistance [6,10,11,12,55,56]. Various pharmacological interventions were used to demonstrate the harmful modulation induced by elevated [Ca^2+^]_i_ on glucose uptake, including the Ca^2+^ ionophore neomycin [12], elevated [K^+^]_e_ [11], and parathyroid hormone [11,55]. To overcome concerns about the multiple side effects of the agents used, we revised this relationship using the heterozygous knock-in mice C57BL/6 for RyR1 (MH-R163C). These mice exhibit a chronic elevation of [Ca^2+^]_i_ without needing pharmacological interventions [21,43]. Using this genetically modified model, we could accurately study the underlying mechanisms and effects of elevated [Ca^2+^]_i_ on the glucose uptake, and eliminated the potential confounding factors introduced by pharmacological treatments. This approach allowed us to conclude that [Ca^2+^]_i_ exerts a biphasic modulation on glucose uptake processes within neuronal cells.

Another important aspect to discuss was the limitations in measuring [Ca^2+^]_i_ in studies that explored the relationship between insulin, [Ca^2+^]_i_, and glucose uptake. Previous studies [47,57,58] relied on BAPTA-based fluorescent Ca^2+^ probes to measure [Ca^2+^]_i_. Fluorescent Ca^2+^ indicators pose significant challenges due to issues such as calibration, probe compartmentalization, leakage, photostability, binding properties, and intracellular Ca^2+^ buffering. In the present study, we overcame the limitations of BAPTA-based fluorescent Ca^2+^ indicators using Ca^2+^-selective microelectrodes with submicrometer tip diameters and a detection limit close to pCa 8, which allowed us a direct and reliable method for measuring and quantifying resting the [Ca^2+^]_i_ in quiescent cells [2,19,31,59]. However, this technique has several limitations, including its invasive nature (cell impalement), dependency on the cell size, and a response time that is too slow to track rapid intracellular Ca^2+^ transients accurately. However, the response time did not interfere with our study, as intracellular Ca^2+^ measurements were conducted in TTX-pretreated quiescent neurons, where rapid transients were not a primary concern.

This observed decrease in the glucose uptake within the MH-R163C neurons represents a concerning scenario, considering the substantial and continuous need for glucose in the brain to meet its increased metabolic demands [60]. This compromised glucose uptake in MH-R163C neurons could significantly alter crucial neurological processes, such as synaptic plasticity, neurogenesis, and cognitive function [60]. Furthermore, exacerbating glucose uptake deficiency in the MH-R163C neurons by isoflurane, a known trigger for MH [3,41], added another layer of concern. This revelation unveiled previously overlooked pathological changes during MH episodes [1], which shed new light on the intricate mechanisms underlying MH syndromes. These findings underscore the need for a comprehensive understanding of the neurological implications of MH and emphasize the importance of addressing metabolic dysregulations alongside well-known muscular manifestations.

### 4.3. Modulation of Glucose Uptake by [Ca^2+^]_i_

This study demonstrated the negative impact of chronic elevated [Ca^2+^]_i_ on insulin-mediated glucose uptake in MH-R163C hippocampal neurons. Furthermore, we showed that pharmacological interventions that lowered the [Ca^2+^]_i_ increased the glucose uptake. Thus, depriving the neurons of extracellular calcium reduced the MH neuronal [Ca^2+^]_i_ to 105 nM and resulted in a significant increase in the insulin-induced glucose uptake by 1.8-fold; SAR734, identified as a TRPC3/TRPC6 channel blocker [24], reduced the [Ca^2+^]_i_ to 100 nM and increased the uptake by 1.9-fold; and Dantrolene, the current first-line treatment for individuals affected by MH [61] and a reduction in [Ca^2+^]_i_ in the muscle cells of patients susceptible to MH [62], swine [27], and mice [2,21,30], as well as cortical and hippocampal neurons [7,23], decreased the [Ca^2+^]_i_ to 95 nM and increased the glucose uptake by 2-fold. Interestingly, these interventions had a minimal impact on the WT neurons, where the [Ca^2+^]_i_ after the pharmacological treatment remained relatively stable around 100 nM (Ca^2+^-free solution: 100 nM, SAR734: 98 nM, and dantrolene: 97 nM).

In contrast, chelating the intracellular calcium with BAPTA, which decreased the [Ca^2+^]_i_ to approximately ≈60 nM in both the MH-R163C and WT neurons, further inhibited aberrant insulin-induced glucose uptake in the MH-R163C neurons but also reduced it in the WT neurons. The physiological level of [Ca^2+^]_i_ (100–130 nM) is vital for facilitating adequate insulin glucose uptake in neurons. Therefore, there is a critical range for [Ca^2+^]_i_ that optimally facilitates insulin-induced glucose uptake, with an upper threshold of approximately 200 nM and a lower limit of 100 nM (estimated using Ca^2+^-selective microelectrodes). This optimal range of neuronal [Ca^2+^]_i_ may offer valuable information on the mechanisms responsible for the impaired glucose transport observed in MH-R163C neurons. Additionally, it emphasizes the potential therapeutic importance of targeting calcium signaling pathways to alleviate metabolic disturbances related to insulin resistance and its associated conditions observed in type 2 diabetic patients.

### 4.4. Intracellular [Ca^2+^] and PI3K/Akt Signaling Pathway

Insulin facilitates glucose uptake and metabolism via the PI3K/Akt pathway. Insulin signaling in the brain is vital in regulating various functions, such as oxidative processes, mitochondrial functions, and neuronal survival [63]. Additionally, emerging evidence suggests that insulin contributes to cognitive functions, such as learning and memory [64]. Furthermore, insulin resistance in the brain is associated with several central nervous system diseases [65].

Insulin receptor activation and consecutive phosphorylation/activation of signaling proteins, mainly PI3K and Akt, are largely involved in insulin action and response in target tissues [66]. Defects in the insulin signaling cascade have been associated with severe insulin resistance and T2D [66]. In our current investigation, we observed a decrease in the expressions of PI3K and AS160 proteins and insulin-induced phosphorylation of pPI3K, pAkt, and pAS160 in the MH-R163C hippocampal neurons compared with the WT neurons. In the MH-R163C hippocampal neurons, there was not only a reduction in the total protein expression but also a decrease in the proportion of protein in its active, phosphorylated form. PI3K/Akt centrally regulates the insulin pathway through the activation of AS160, which plays a critical role in the modulation of glucose transporter GLUT4 trafficking [66]. This finding agrees with the changes previously observed in skeletal muscles from MH-R163C and db/db mice [10].

Another interesting finding was the alteration of the phosphorylated/total protein ratio in the PI3K/Akt and AS160 in the MH-R163C hippocampal neurons compared with the WT neurons. This ratio is a valuable biomarker that reflects the activation status of the insulin signaling pathway, which is crucial to regulating the glucose uptake in insulin-targeted cells. A decrease in the phosphorylated/total protein ratio, as observed in the MH-R163C neurons (Figure 9), indicates reduced pathway activation, which can alter the glucose uptake and contribute to insulin resistance. The impaired ability of neurons to respond to insulin not only disrupts the glucose metabolism but has also been linked with neurodegenerative conditions or cognitive impairments in T2D patients [67,68].

Three isoforms of the GLUT family (GLUT1, GLUT3, and GLUT4) have been detected in regions of the brain, including the basal forebrain; hippocampus; sensorimotor cortex; hypothalamus; and, to a lesser extent, the cerebral cortex and cerebellum [69]. Functionally, GLUT4 is involved in insulin-dependent glucose transport, which is predominantly expressed in insulin-sensitive tissues, such as skeletal muscle, adipose tissue, and the brain, more notably in the hippocampus [57]. In the present study, GLUT4 expression was significantly reduced in the MH-163C hippocampal neurons compared with the WT mice. The decrease in the GLUT4 expression is consistent with our previous findings in skeletal muscle from MH-R163C and db/db mice [10], suggesting a possible connection to the observed decline in neuronal glucose uptake. While insulin resistance has traditionally been studied in metabolic tissues, such as muscle and adipose tissue, emerging evidence indicates that it also occurs in the brain [63].

Based on previous studies [6,10,70] and current data, it is compelling to propose that the intracellular calcium concentration may modulate the PI3K-Akt signaling pathway, as well as GLUT4 expression and translocation, and consequently regulate neuronal glucose uptake.

### 4.5. Glucose Uptake, Insulin, and Hippocampal Memory

Emerging evidence indicates that insulin, in addition to facilitating glucose uptake, plays a role in the cognitive processes of the hippocampus [64]. Conditions that compromise brain glucose supply or transport, such as aging, T2D, and Alzheimer’s disease, have been associated with impaired cognitive performance [65,71,72]. In this context, GLUT4 is essential for addressing insulin resistance in the brain and preventing cognitive memory dysfunction [73,74]. Although the precise mechanism by which GLUT4 dysfunction affects cognitive function remains unclear, our study highlighted an association between the hippocampal insulin resistance and the reduced expression of GLUT4 in MH-R163C neurons. These findings suggest that individuals susceptible to MH may experience cognitive impairment.

### 4.6. Study Limitations

Although our study was pioneering in unveiling, for the first time, glucose dyshomeostasis in MH-R163C pyramidal hippocampal neurons, it is important to recognize several limitations: (i) Although female and male mice were included in the experimental group, sex was not examined in detail as a potential source of variability in the intracellular Ca^2+^ levels and glucose dyshomeostasis due to the limited sample size. (ii) We did not explore whether the genetic manipulation of RyR and/or TRPC channels could rescue or alleviate the reduced glucose uptake observed in the MH-R163C neurons. (iii) The impact of reducing the [Ca^2+^]_i_ on the expression of the PI3K/Akt signaling pathway was not investigated. (iv) Mechanisms by which the [Ca^2+^]_i_ modulated the global expression of GLUT4, and the extent of GLUT4 phosphorylation in the MH-R163C neurons, were not studied.

### 4.7. Summary

This study demonstrated that [Ca^2+^]_i_ played a modulatory role in neuronal glucose uptake. A reduction in the [Ca^2+^]_i_ below the physiological level led to a significant decrease in the insulin-stimulated glucose uptake in both genotypes examined. In contrast, chronic elevations in the [Ca^2+^]_i_, as seen in the MH-R163C neurons, significantly reduced the insulin-stimulated glucose uptake. The relationship between the [Ca^2+^]_i_ and the insulin response suggests that both low and high [Ca^2+^]_i_ levels impaired the neuronal sensitivity to insulin. Additionally, the exposure of the MH-R163C neurons to isoflurane further increased the [Ca^2+^]_i_, which worsened the already compromised insulin-dependent glucose uptake. Moreover, hippocampal homogenates from the MH-R163C mice showed changes in the PI3K/Akt signaling pathway and GLUT4 expression. Elucidating these mechanisms provides insight into the complexity of insulin signaling at the cellular level. By identifying the pathways contributing to insulin dysfunction, more effective strategies for preventing and managing metabolic disorders, such as T2D, can be developed.

## 5. Conclusions

The intracellular calcium concentration played a pivotal role as a modulator in the signaling cascade between the insulin-activated receptor complex on the plasma membrane and the neuronal GLUT4-mediated glucose uptake.

## Data Availability

The datasets generated and analyzed during the current study are available from the corresponding author upon request.

## Supplementary Materials

The following supporting information can be downloaded at: https://www.mdpi.com/article/10.3390/cells13221888/s1, Figure S1: Representative Records of intracellular Ca^2+^ concentration and insulin glucose uptake in WT and MH-R163C single neurons; Figure S2: Sarcoendoplasmic Ca^2+^ Loading.
