# Supplementary material for: Impairment of Glucose Uptake Induced by Elevated Intracellular Ca2+ in Hippocampal Neurons of Malignant Hyperthermia-Susceptible Mice
Source: Cells. 2024 Nov 15;13(22):1888. doi: 10.3390/cells13221888 (PMC11592500; doi:10.3390/cells13221888)
Supplement: Supplementary file 1 [file cells-13-01888-s001.zip › Supplemental Figure 1.pdf]

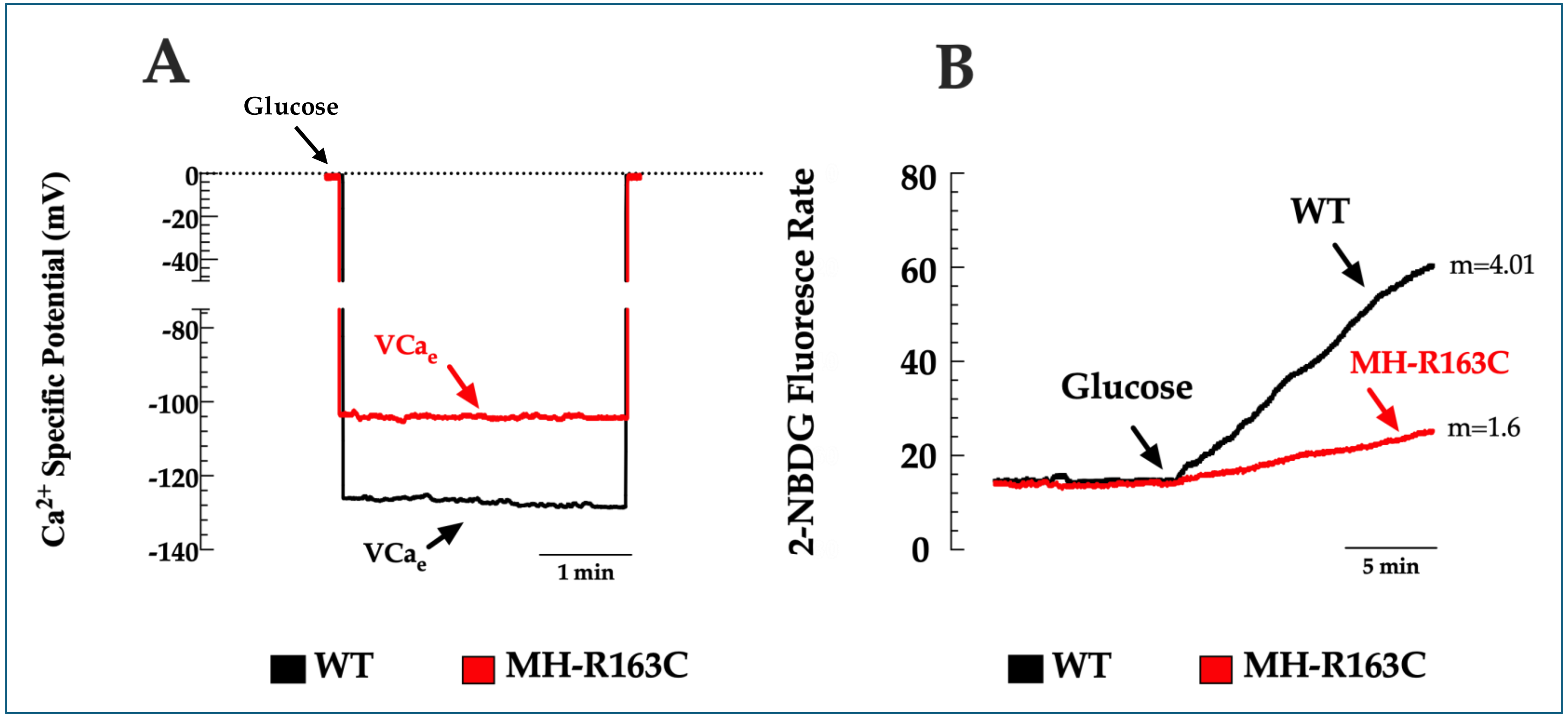

Supplemental Figure 1. Representative Records of intracellular Ca^{2+} concentration and insulin glucose uptake in WT and MH-R163C single neurons. A. Representative calcium-specific potential (VCa_e) traces measured in WT and MH-R163C hippocampal neurons using Ca^{2+}-selective microelectrodes. The intracellular $[Ca^{2+}]_i$ in the WT neuron was 119 nM, while in the MH-R163C neuron, it was significantly elevated to 320 nM, indicating impaired intracellular Ca^{2+} regulation in MH-susceptible MH-R163C neurons. **B**. Representative glucose uptake measurements in WT and MH-R163C neurons using 2-NBDG reveal a 2.2-fold reduction in glucose uptake in MH-R163C neurons compared to WT neurons, indicating a compromise insulin-dependent glucose uptake in MH-R163C. ***m*** represents the slope values for each condition; calibration bars**:** 1 minute for VCa_e recording and 5 minutes for glucose uptake.
