# Supplementary material for: Impairment of Glucose Uptake Induced by Elevated Intracellular Ca2+ in Hippocampal Neurons of Malignant Hyperthermia-Susceptible Mice
Source: Cells. 2024 Nov 15;13(22):1888. doi: 10.3390/cells13221888 (PMC11592500; doi:10.3390/cells13221888)
Supplement: Supplementary file 1 [file cells-13-01888-s001.zip › Supplemental Figure 2.pdf]

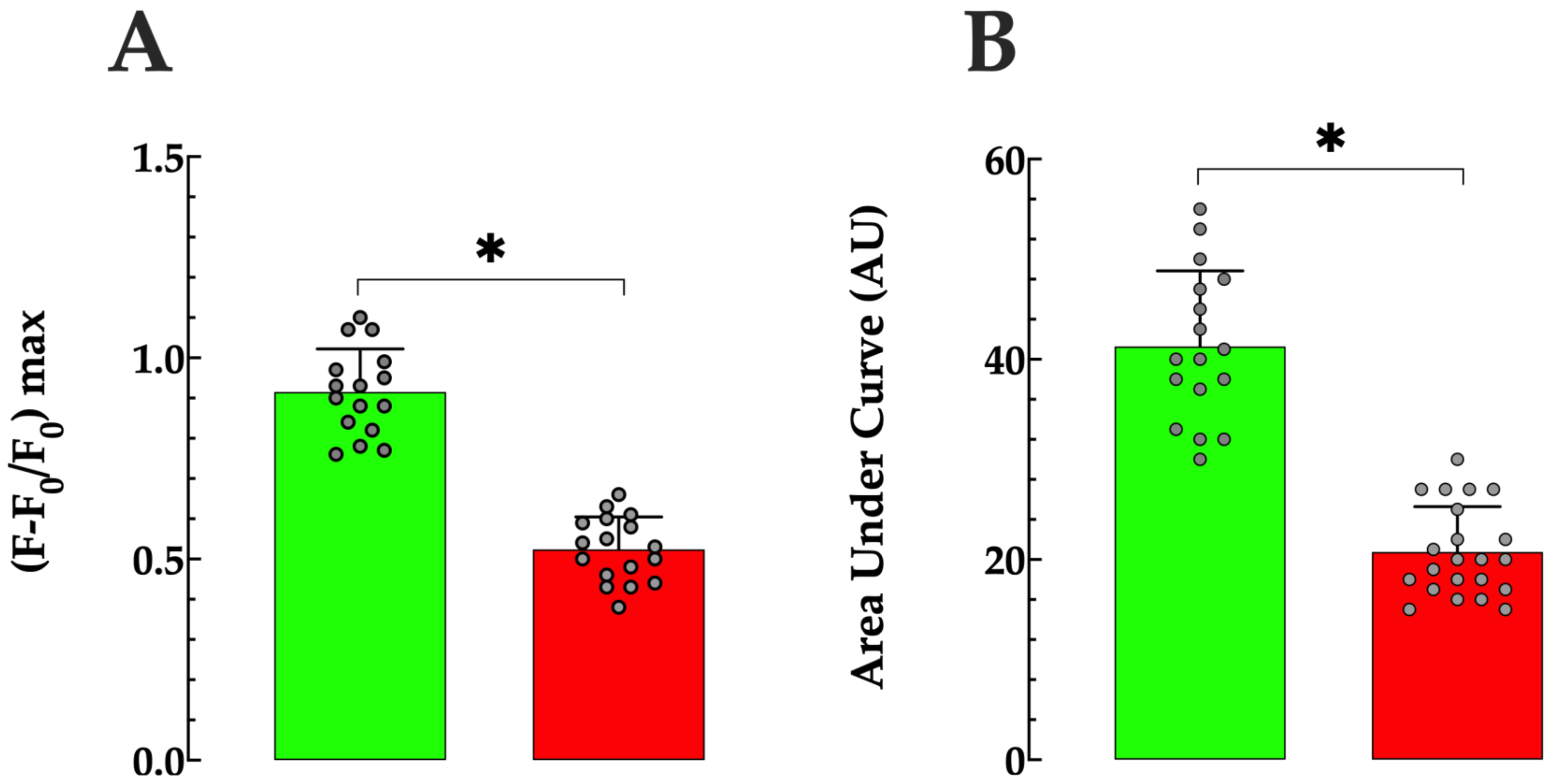

Supplemental Figure 2. Sarcoendoplasmic Ca^{2+} Loading. WT and MH-R163C hippocampal neurons were loaded with 5 µM Fluo-4 AM for 30 minutes at 37°C. then, the neurons were incubated in nominally Ca^{2+}-free Ringer's solution supplemented with 2 mM EGTA. To induce Ca^{2+} release from the sarcoendoplasmic reticulum (SER), a Ringer's solution containing 5 µM ionomycin was applied, and the resulting Ca^{2+} transient was quantified. The Ca^{2+} signal was monitored using excitation and emission wavelengths of 480/30 nm and 535/40 nm, respectively. A. Quantification of peak transient amplitude showed a 1.7-fold reduction in MH-R163C neurons (n=16) compared to WT neurons (n=17). B. The integrated area of ionomycin-induced Ca^{2+} transients in hippocampal neurons indicated that the SER Ca^{2+} store content was significantly lower in MH-R163C neurons than in WT neurons. The integrated area in MH-R163C hippocampal neurons was 21 ± 4 arbitrary fluorescence units (AU) (n=22), compared to 41 ± 7 AU in WT neurons (n=17, $p < 0.05$, t-test). This observation supports the hypothesis of an increased resting SER Ca^{2+} leak in MH-R163C hippocampal neurons, which may lead to reduced SER Ca^{2+} loading. This may also result in the upregulation of TRPC3 and TRPC6, causing increased Ca^{2+} influx through these voltage-independent calcium channels.
